# Pepper Constituents Enhance the Toxicity and Neurophysiological Effects of Natural Pyrethrins in Insects

**DOI:** 10.3390/insects17050510

**Published:** 2026-05-17

**Authors:** Edmund J. Norris, Jeffrey R. Bloomquist

**Affiliations:** 1 United States Department of Agriculture, Center for Medical, Agricultural, and Veterinary Entomology, Gainesville, FL 32608, USA; 2 Department of Entomology and Nematology, Emerging Pathogens Institute, University of Florida, Gainesville, FL 32610, USA; jbquist@epi.ufl.edu

**Keywords:** lipid alkamides, pyrethrins, *Aedes aegypti*, synergism

## Abstract

Control of resistant mosquito populations may be approached by the development of novel synergists to improve the performance of already commercialized compounds. Extracts of black pepper (*Piper nigrum*), Cha Plu (*Piper sarmentosum*), and Sichuan pepper (*Zanthoxylum* spp.) synergized natural pyrethrins applied topically to *Aedes aegypti* females. Both black pepper and Sichuan pepper extracts synergized natural pyrethrins over 13-fold. Synergism was also observed directly on the mosquito larval central nervous system (CNS), suggesting this effect is an important contributing factor distinct from that of reduced metabolism. Piperine, from black pepper, and α-hydroxysanshool (α-HS) from Sichuan pepper, synergized natural pyrethrins on susceptible CNS, but only piperine was capable of synergizing natural pyrethrins on the CNS of a pyrethroid-resistant strain of *Ae. aegypti*, indicating that these molecules may possess slightly different mechanisms of action or binding sensitivities. These results demonstrate that pepper alkamides are capable of enhancing select insecticidal chemistries via target-site synergism, a novel mechanism of synergism that might be important for enhancing future insecticidal formulations.

## Simple Summary

Voltage-gated sodium channels are the primary target site for many insecticides and natural products, including natural pyrethrins and pyrethroids. Mutations in these channels can reduce insecticide sensitivity and contribute to resistance. Previous work has shown that a select class of compounds, lipid alkamides, are able to affect sodium channels via a distinct mechanism from pyrethroids. Due to these distinct characteristics, it is possible that lipid alkamides may synergize natural pyrethrins or other sodium channel-directed insecticides. This study evaluated the potential of lipid alkamides to synergize natural pyrethrins. We found they are capable of synergizing natural pyrethrins via target-site synergism; however, these effects are mitigated in at least some forms of kdr-resistance.

## 1. Introduction

As resistance to insecticides continues to develop, particularly in populations of disease vectors, the development of new control technologies will increase in importance (Nauen 2007) [1]. Resistance in mosquito populations is common worldwide, resulting in the loss of insecticide effectiveness (Liu 2015; Haddi et al., 2017) [2,3]. It is possible to circumvent insecticide resistance through rotation of compounds with different modes of action or using metabolism-blocking synergists (Darriet & Chandre 2011; Somwang et al., 2011; Gao et al., 2012; Yu 2015) [4,5,6,7]. However, these methods may not always fully overcome resistance, depending upon the mechanisms present and their genetic inheritance. For example, mono-oxygenase inhibitors such as piperonyl butoxide (PBO) can effectively reverse resistance in insects with upregulated oxidase metabolism, but this strategy is ineffective against strains displaying target-site resistance (Brooke et al., 2001; Nwane et al., 2013) [8,9].

**Figure 1:**
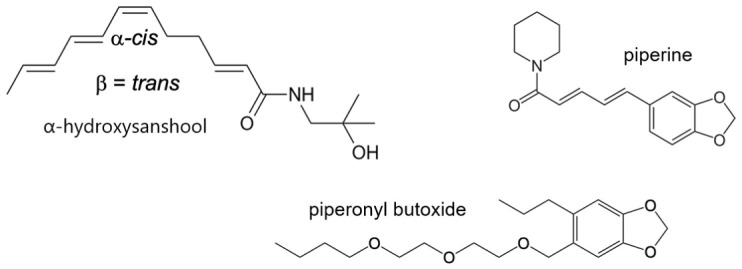
Chemical structures of piperonyl butoxide and the major components of the extracts used or discussed in this study.

There has been increased interest in natural materials for insect control, including specific botanical compounds and essential oil mixtures (Isman 2000) [10]. Black pepper extracts and the component compounds piperine (Figure 1) and pellitorine have been identified as insecticides and synergists (Scott et al., 2007) [11]. These compounds are of the lipid amide chemical class and act as sodium channel agonists via interaction at the alkaloid binding site that also recognizes other plant toxins, such as veratridine and aconitine (Ottea et al., 1989, Bloomquist, 1996) [12,13]. Further, the alkaloid binding site on the voltage-gated sodium channel is coupled allosterically to the pyrethroid-binding site (Jacques et al., 1980; Bloomquist & Soderlund 1988) [14,15]. This mechanism underlies the synergistic effect of alkaloid + pyrethroid mixtures, both at the level of the nerve and in lethality studies (Norris and Bloomquist 2022) [16]. This pharmacological coupling has not received much study in either wild type or kdr insect strains. However, several studies have shown aconitine cross resistance in kdr housefly (Salgado et al., 1983) [17], cockroach (Dong and Scott, 1991) [18], and mosquito (Norris and Bloomquist, 2022) [16] strains. Norris and Bloomquist (2022) [16] further reported that aconitine lost its synergizing effect of natural pyrethrins when tested on the kdr-expressing Puerto Rico strain of *Ae. aegypti*.

Early work showed that the lipid amides were relatively unaffected by the kdr mutation (L1014F) found in houseflies (Elliot et al., 1986; Du, Khamby & Dong 2011) [19,20] and therefore might be excellent candidate target site synergists of pyrethroids and perhaps other insecticides. Accordingly, the present study further explored the synergistic potential of pepper extracts and their predominant derived lipid alkamides when applied in combination with natural pyrethrins (NPs) against the yellow fever mosquito, *Aedes aegypti.* Evaluation of compound performance was assessed by in vivo exposure to adults, as well as in vitro via direct application to the mosquito larval CNS and cockroach giant axon systems. Effects were characterized on both pyrethroid-resistant (Puerto Rico) and pyrethroid-susceptible (Orlando) strains. The resistance mechanisms of the Puerto Rico strain provide high levels of resistance to DDT and pyrethroids (Estep et al., 2017) [21] and include a low level of elevated monooxygenase, with the major component being kdr mutations at Val1016Iso and Phe1534Cys. Overall, it was expected that the results would aid in understanding how kdr resistance might be overcome by mixtures of different compounds with pyrethroids and could aid in the design of synergizing mixtures for control of mosquito populations.

## 2. Materials and Methods

### 2.1. Chemicals

Black pepper (*Piper nigrum*) and Sichuan pepper (*Zanthoxylum* spp.) were sourced from Tones (Ankeny, IA, USA) and Natural Green Plus (Queens, NY, USA), respectively. Extracts were obtained via agitation of 10 g of each raw pepper sample in a simple extraction solution of 1:1 solution of ethyl acetate:methanol for 15 min. Raw material was filtered using Whatman filter No. 2. Solvent was allowed to evaporate slowly in a chemical fume hood, and raw sample was then collected and maintained in the refrigerator. Piperonyl butoxide, a semi-synthetic analog of safrole found in sesame oil [22], was purchased from Sigma-Aldrich (St. Louis, MO, USA). The sample of natural pyrethrins (NPs) was from Fairfield American Corporation, (Frenchtown, NJ, USA). For comparison with the in vitro studies, NP molecular weight was estimated using the average molecular weight of all six pyrethrin components and their proportion within the sample (52%). Piperine from black pepper and α-hydroxysanshool (α-HS) from Sichuan pepper, having purities of 98% and >95%, were obtained from Sigma Aldrich (St. Louis, MO, USA) and Chengdu Alfa Biotechnology Co., Ltd. (Chengdu, China), respectively. All compounds were dissolved in 100% ethanol (from Sigma Aldrich) as a vehicle for topical application to adult mosquitoes. Inorganic salts and HEPES buffer for physiological salines were obtained from Fisher Scientific (Hampton, NH, USA).

### 2.2. Mosquitoes and Bioassays

Aedes aegypti mosquito larvae of the susceptible Orlando and pyrethroid-resistant Puerto Rico strains were obtained from the United States Department of Agriculture Center for Medical, Agricultural, and Veterinary Entomology. Larvae were provided 3:1 liver powder and yeast daily and maintained at 28 °C and 40–50% humidity. As pupae appeared, they were separated from the larvae and allowed to eclose in separate rearing cages. Adults were provided 10% sugar water ad libitum until bioassays were performed. Mosquitoes (2–5 days old) were aspirated from colony cages and cold anesthetized on ice for use in bioassays. Females were hand sorted and given doses of pyrethrins, synergists, or combinations thereof via micro-application to the pronotum. A repeating applicator delivering 0.2 µL of solution was used for topical treatments via Hamilton gas-tight syringes. All synergist candidates were tested at sublethal levels with NP (500 ng/insect for the pepper extracts and lipid alkamides, and 2000 ng/insect for PBO). PBO was added in all synergistic mixtures to account and mitigate the potential for metabolic degradation of the candidate target-site synergists. After treatment, knockdown and mortality were recorded at 1 h and 24 h post-application, respectively. At least 10 mosquitoes were utilized for each concentration tested, representing a single replicate, and at least 3 distinct rearing cohorts (reared from separate egg batches) were used for each concentration screened. The treated mosquitoes were then transferred to a 16-ounce deli cup with tulle fabric placed over the top to prevent escape. Mosquitoes were then transferred to an incubator and maintained at a constant temperature of 28 ± 2 °C with a light cycle of 12:12 h light: dark. The humidity was maintained at a relatively constant 75 ± 10% RH using a water pan placed at the bottom of the incubator. Only non-blood-fed mosquitoes were used in the assays. A minimum of four concentrations were used for each dose–response curve for each treatment.

### 2.3. Electrophysiology

Nerve firing from the central nervous system of third or fourth instar *Ae. aegypti* larvae was recorded using techniques described recently by Norris and Bloomquist (2022) [16]. Briefly, larvae were dissected in a small wax-lined dish to provide access to the abdominal nerve cord and the preparation bathed in physiological saline containing (mM) NaCl (175), KCl (3), CaCl2 (2), HEPES (4), pH 7.2. The connectives were severed using forceps and a suction electrode was attached within the connective opening. A differential amplifier in AC coupled mode (Model 3000, A-M Systems, Inc., Carlsborg, WA, USA) recorded action potentials and a Humbug (Quest Scientific, North Vancouver, BC, Canada) suppressed 50–60 Hz noise. Signals were then digitized, amplitude-discriminated with a threshold just above background noise, and converted to frequency (Hz) using LabChart Pro 7 (AD Instruments Inc. Colorado Springs, CO, USA). Baseline electrical activity was determined for 5–10 min prior to drug application. Each preparation was monitored for 30 min, and fresh CNS preparation was used for each treatment and replicate. A minimum of three replicates (individual larvae in each replicate) was used for every concentration of drug.

Resting membrane and action potentials from *Periplaneta americana* giant axons, in situ, were performed as described by Jiang and Bloomquist (2021) [23]. The recording chamber contained a small glass platform filled with saline of the following composition (mM) NaCl (210), KCl (3), CaCl2 (2), and HEPES (4), pH 7.2. An excised abdominal nerve cord was stretched over the platform, where a small region (ca. 0.5 mm) of the connectives between the fourth and fifth ganglia was manually desheathed with fine forceps. A suction glass electrode was attached to the nerve cord at the second abdominal ganglion, and rectangular stimuli of 0.1–3 V were provided by a Grass S9 stimulator (Grass Instruments, Quincy, MA, USA). A recording microelectrode of 15–30 MΩ resistance was then inserted into the desheathed region of the nerve cord. An AxoClamp 2B (Molecular Devices, San Jose, CA, USA) was used to record resting and action potentials, which were digitized and analyzed with LabChart 8 Pro software (ADInstruments, Colorado Springs, CO, USA). All impalements were allowed to stabilize for 10 min prior to treatment. Compounds were dissolved in DMSO and 1 µL was added to the saline bath and mixed by hand pipetting. Treatments were allowed to incubate for 1 min before recording resting membrane potentials or action potentials. A train of continuous stimuli at 10 Hz were provided after drug application to activate channels and screen for use-dependent effects. Each treatment was replicated at least three times, using a new cockroach nerve cord preparation for each replicate.

### 2.4. Data Analysis

PROC PROBIT (SAS version 9.4, SAS Institute, Cary, NC, USA) was used to determine LD50 values, along with the 95% confidence limits (95% CL). Synergist ratio was calculated via the following calculation: SR = LD50 NP/LD50 NP + PBO (or extract in BOLD). The pepper-specific contribution to synergism was calculated via the following: SR = SR with PBO + Pepper/SR with PBO alone in ternary treatments assuming multiplicative action. Larval CNS firing frequency was averaged over 1 min intervals, immediately prior to the application of the tested compound (baseline) and every 1 min thereafter. Mean firing rates of control and drug treatments on larval CNS were subjected to an unpaired t-test between to determine statistical significance at every time point compared to the DMSO controls (α = 0.05). To determine if significant block or hyperexcitation occurred in treatments compared to the DMSO (vehicle) control, each timepoint within each treatment was evaluated for significant differences compared to the control. For cockroach nerve cord preparations, differences in spike parameters between treatments were assessed using unpaired t-tests at discrete concentrations and times.

## 3. Results

### 3.1. Adult Topical Toxicity

Combinations of pepper extracts and PBO produced varying levels of synergism of NP, depending on the strain of mosquito under study (Table 1). PBO alone was an effective synergist, as expected, and the results show that adding BPE or SPE to PBO treatments enhanced the synergistic activity in the susceptible Orlando strain. Moreover, the extract contribution equals or exceeds that of PBO alone when compared at both the LD_50_ and LD_90_ levels, assuming the effects of the two synergists were purely multiplicative (i.e., independent synergism beyond that provided by PBO alone). This finding was also true for piperine and to a lesser degree with α-HS (Table 1). On the pyrethroid-resistant strain, the PBO SR value was increased 42% (at the LD_50_ level), but the synergist ratios when adding the pepper extracts or components were reduced compared to their specific contribution to synergism on the susceptible strain (Table 1). In particular, both SPE and α-HS did not significantly improve the efficacy of NP beyond that of PBO (as indicated by significant overlap in the 95% CI compared to NP + PBO alone), indicating that little-to-no synergism beyond that already produced by mono-oxygenase inhibition was present. This was particularly noteworthy at the LD_90_ level, with a synergism ratio of just the SPE and α-HS alone (after removing the PBO contribution) being significantly lower than 1.

### 3.2. Larval CNS Recordings

**Figure 2:**
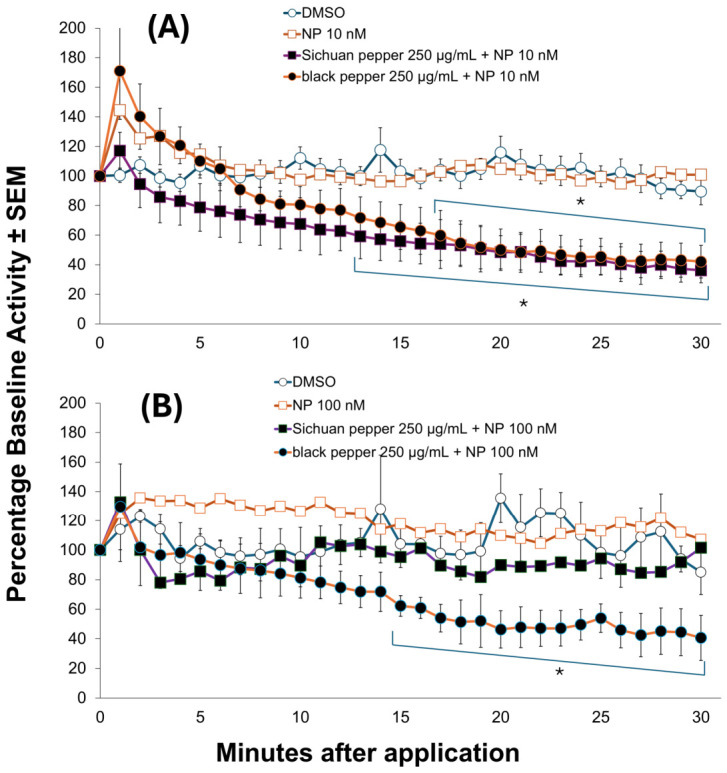
Synergism of natural pyrethrins (NP) by Sichuan and black pepper extracts in susceptible mosquito larval CNS ((**A**) top) and pyrethroid-resistant ((**B**) bottom). Statistical significance observed is denoted with an asterisk (*) (*p*-value < 0.05). Sichuan pepper and black pepper extracts synergized NP in the pyrethroid-susceptible Orlando strain, but only black pepper extract synergized NP in the pyrethroid-resistant Puerto Rico Strain. Select error bars were omitted for natural pyrethrins (NP) treatments for clarity.

It was demonstrated in our previous study (Norris and Bloomquist, 2022) [16] that on the Orlando strain, 10 nM NP did not produce blockage of nervous system firing for the entire duration of the experiment (30 min); however, it did produce significant but incomplete nerve blockage (ca. 60% at 30 min) at 100 nM. Thus, 10 nM of NP was tested with the extracts in the present study on the pyrethroid-susceptible strain. When combined with BPE or SPE at concentrations that alone did not produce significant excitation/block compared to the vehicle control, the combination treatments showed synergism of NP effects at the level of the nerve, taking the form of enhanced nerve block (Figure 2). Specifically, concentrations of the extracts (250 ppm) that were inactive alone significantly enhanced nerve block by 10 nM NP on the susceptible strain (ORL). Previous experiments [16] demonstrated that 100 nM NP did not produce blockage of nervous system firing in the pyrethroid-resistant Puerto Rico strain, and as such it was used as the screening concentration to evaluate NP synergism by pepper extracts. Again, 250 µg/mL of each extract did not produce significant nerve hyperexcitation or block in the Puerto Rico strain, and was used as the screening concentration to evaluate synergism of NP. Interestingly, only black pepper extract was capable of producing significant effects, with nerve block observed at later time points when coapplied with NP 100 nM.

**Figure 3:**
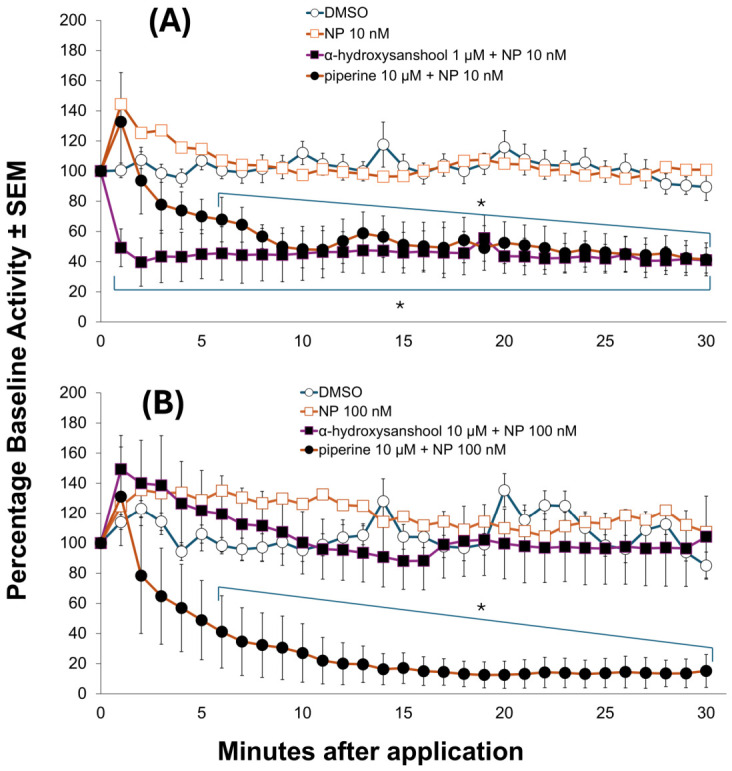
Synergism of natural pyrethrins (NPs) by α-HS and piperine in susceptible mosquito larval CNS ((**A**) top) and pyrethroid-resistant strain ((**B**) bottom). Statistical significance observed is denoted with an asterisk (*) (*p*-value < 0.05). α-HS and piperine synergized NP in the pyrethroid-susceptible Orlando strain, but only piperine synergized NP in the pyrethroid-resistant Puerto Rico Strain. Select error bars were omitted for natural pyrethrin (NP) treatments for clarity.

Additional experiments characterized the synergistic interactions of α-HS and piperine in combination with NP on the susceptible and pyrethroid-resistant strains (Figure 3). Initial screening of the predominant lipid alkamides in each extract was performed at 10 µM for both strains (Supplemental Figure S1). Piperine did not produce significant block in either strain at 10 µM, and thus this concentration was used for subsequent synergism screening. Differences were observed, however, for α-HS across between strains. While 10 µM of αHS did not produce block in the pyrethroid-resistant strain, it did produce significant block in the pyrethroid-susceptible strain (ORL). Therefore, while 10 µM of αHS was used as the synergist screening concentration for the pyrethroid-resistant strain, a lower dose of 1 µM of α-HS was used in the pyrethroid-susceptible strain as it did not produce statistically significant effect on its own in this strain. These experiments showed high potency effects of these constituents, similar to the whole extracts screened previously. Both single constituents from black pepper and Sichuan pepper enhanced the nerve block produced by NP 10 nM in the pyrethroid-susceptible strain. Unexpectedly, only piperine (10 µM) enhanced the block of 100 nM NP on the pyrethroid-resistant strain, mirroring results we observed with BPE and SPE. The higher concentration of α-HS (10 µM) screened on the pyrethroid-resistant strain did not significantly enhance NP effects.

### 3.3. Cockroach Giant Axon Recordings

For comparison and to assess effects on the cellular nerve level, an exploratory screen (one biological replicate) of piperine and α-HS were tested on American cockroach giant axons. Piperine at 1 µM had no effect, but at 10 µM there was a progressive reduction in the action potential amplitude of about 32% (Supplemental Figure S2). There was also a broadening of the action potential duration, which was increased 71%. The other plant alkaloid, α-HS, produced a similar but distinctive set of effects. For instance, α-HS did produce a reduction in the action potential amplitude, and it depolarized the nerve, particularly at higher concentrations. These results may indicate that both alkaloids uniquely interact with axonal ion channels.

## 4. Discussion

The present study demonstrates that pepper-derived extracts and their constituent lipid amides can act as effective synergists of natural pyrethrins (NPs), but that the magnitude and consistency of this effect is strongly dependent on the resistance background of the mosquito strain under study. In the pyrethroid-susceptible Orlando strain, BPE, SPE, piperine, and α-HS all substantially enhanced NP toxicity, often exceeding the level of synergism provided by PBO alone. In contrast, synergism in the pyrethroid-resistant Puerto Rico strain was markedly attenuated, with piperine and BPE retaining modest activity, whereas SPE and α-HS contributed little or no additional benefit beyond that provided by PBO. These findings underscore the importance of target-site resistance mechanisms in shaping the effectiveness of putative synergists and provide insight into why certain botanical additives perform well in susceptible populations yet may fail to overcome kdr-based resistance.

In the Orlando strain, the strong synergism observed with both whole extracts and isolated lipid amides is consistent with the established pharmacology of these compounds as sodium channel agonists that bind at the site 2 alkaloid binding domain (Ottea et al., 1989; Bloomquist 1996) [12,13]. This site is positively allosterically coupled to the pyrethroid-binding site on the voltage-gated sodium channel, providing a mechanistic basis for enhanced toxicity when lipid amides and pyrethrins are applied together (Jacques et al., 1980; Bloomquist and Soderlund 1988) [14,15]. The adult topical bioassay data clearly reflect this interaction, as ternary mixtures containing NP, PBO, and pepper extracts yielded synergist ratios that exceeded those predicted from PBO alone, indicating that the pepper-derived components could be contributing pharmacodynamically/pharmacokinetically rather than simply through metabolic inhibition.

The larval CNS electrophysiology experiments support a direct neural mechanism underlying this synergism. Concentrations of BPE, SPE, piperine, and α-HS that were inactive when applied alone significantly enhanced NP-induced nerve block in the susceptible strain, demonstrating that these compounds can amplify pyrethroid effects at the level of neuronal excitability. This observation aligns with previous work showing that lipid amides may enhance pyrethroid-induced channel dysfunction by prolonging channel open times or promoting use-dependent block [24] (Norris and Bloomquist 2022) [16]. Importantly, the close correspondence between adult toxicity and larval CNS responses strengthens the inference that the observed synergism in vivo is driven primarily by sodium channel-level interactions.

In contrast, the diminished synergism observed in the Puerto Rico strain highlights the constraining influence of kdr mutations on site-specific pharmacological interactions. This strain harbors Val1016Ile and Phe1534Cys substitutions [21], which are known to reduce pyrethroid sensitivity and alter channel gating properties (Chen et al., 2019) [25]. Under these conditions, PBO remained an effective synergist, consistent with the presence of some metabolic resistance, but the incremental benefit of pepper extracts and α-HS was substantially reduced. This pattern mirrors earlier reports of cross-resistance between kdr mutations and site 2 alkaloids such as aconitine in houseflies, cockroaches, and mosquitoes (Salgado et al., 1983; Dong and Scott 1991; Norris and Bloomquist 2022) [16,17,18]. Together, these findings suggest that structural or conformational changes associated with kdr mutations limit the functional coupling between the alkaloid and pyrethroid binding sites, thereby reducing the capacity for target-site synergism.

Notably, piperine differed from α-HS and SPE in that it retained measurable synergistic activity in the resistant strain at both the adult and larval CNS levels. One explanation for this behavior is that piperine may act through multiple mechanisms. In addition to its activity as a sodium channel agonist, piperine is known to inhibit cytochrome P450 monooxygenases (Scott et al., 2008) [11], raising the possibility that its residual synergism reflects a combination of metabolic inhibition and direct channel modulation. It is possible that piperine is capable of inhibiting distinct cytochromes P450 that are not partially/fully inhibited by PBO. Other groups have seen that PBO is not capable of completely rescuing susceptibility, even in strains that are resistant through metabolic mechanisms (Smith et al., 2018; Zhou et al., 2022; Syme et al., 2022) [26,27,28]. Although PBO is generally considered non-selective, these studies suggest the inhibitory effectiveness of PBO can vary across CYP isoforms.

The cockroach giant axon recordings provide additional insight into mechanistic differences among the lipid amides tested. Piperine reduced action potential amplitude and markedly broadened action potential duration, consistent with altered sodium channel kinetics. In contrast, α-HS produced depolarization and reductions in spike amplitude that suggest a somewhat different interaction with these channels. These distinct electrophysiological signatures indicate that, despite their shared classification as lipid amides, piperine and α-HS do not act identically at the cellular level. Such differences could also contribute to their divergent performance as synergists in kdr-resistant mosquitoes and emphasize the need to evaluate individual constituents rather than assuming uniform activity across botanical extracts. These two differences (i.e., ability to inhibit cytochromes and act on sodium channels in a distinct way compared to α-HS) may allow piperine to partially compensate for reduced target-site sensitivity in kdr-expressing mosquitoes.

We observed differential sensitivity of the pyrethroid-resistant strain to α-HS compared to the pyrethroid-susceptible strain. On the pyrethroid-susceptible strain, we evaluated synergism at a lower dose (1 µM) and a higher dose on the pyrethroid-resistant strain (10 µM). At 1 µM, α-HS produced significant block on the pyrethroid-susceptible strain, but equivalent effect required 10 µM on the pyrethroid-resistant strain. Thus, it is possible that the resistance to α-HS is partially/completely driving the lack of synergism observed in the pyrethroid-resistant strain. We observed a similar phenomenon in a previous study [16], where aconitine synergized NP only on the pyrethroid-susceptible Orlando strain and did not synergize the pyrethroid-resistant Puerto Rico strain, which also exhibited cross resistance to aconitine. It is well established α-HS in mammals modulates somatosensory excitability through inhibition of background (two-pore domain) potassium channels (e.g., KCNK/TASK and TREK families), leading to membrane depolarization and the characteristic tingling/paresthetic sensation [29]. Concomitant activation or sensitization of transient receptor potential channels (notably TRPA1 and TRPV1) has also been reported, further enhancing neuronal firing and sensory signaling [30,31]. This depolarizing, excitatory profile in mammalian neurons aligns closely with (but is distinct from) our electrophysiological observations in insects, where α-HS reduced action potential amplitude and depolarized the resting membrane potential, indicating disruption of ion channel homeostasis at the axonal level. However, in contrast to the canonical mammalian emphasis on K/TRP channel modulation, our study demonstrates that α-HS participates in sodium channel-linked target-site interactions in insects, likely via allosteric coupling between alkaloid and pyrethroid binding domains [12,13,16], thereby enhancing pyrethrins-induced channel dysfunction in susceptible strains. The diminished efficacy of α-HS in the kdr-resistant strain further suggests that, while its depolarizing effects may be broadly conserved across taxa, its contribution to synergism in insects is contingent upon sodium channel architecture (which is altered in kdr-resistant insects [20]), highlighting a divergence between primary sensory mechanisms in mammals and insecticidal target-site interactions observed here.

From an applied perspective, the results of this study suggest that pepper-derived synergists have the greatest potential in populations where pyrethroid susceptibility is retained. In contrast, their utility against strongly kdr-based resistant populations is reduced, but select molecules in this class could be capable of circumventing or limiting resistance. These findings reinforce the broader conclusion that no single synergist is likely to overcome all resistance mechanisms and that effective resistance management will require careful matching of synergist chemistry to the underlying resistance profile of the insecticide and target population (Brooke et al., 2001; Nwane et al., 2013) [8,9].

## 5. Conclusions

Overall, this work advances our understanding of how botanical lipid amides interact with pyrethrins at both organismal and neuronal levels and demonstrates how these interactions are preserved or disrupted in a single kdr-resistant strain. The combined use of adult bioassays and in vitro electrophysiology provides a robust framework for dissecting synergist mechanisms and should prove useful for screening future candidate compounds. With continued research, it may be possible to identify distinct chemistries that are capable of synergizing various insecticides on wild populations, even those that are highly resistant to currently available synthetic insecticides. Robust screening and a better understanding of the toxicodynamics of these chemistries may lead to next-generation formulations that target specific resistance classes, thus reducing the scourge of insecticide resistance.

## Figures and Tables

**Table 1:** Evaluation of natural pyrethrins (NP) synergism studies in topical applications to *Ae. aegypti* adult females in combination with piperonyl butoxide (PBO), black pepper extract (BPE), piperine, Sichuan pepper extract (SPE), or α-hydroxysanshool (α-HS). Synergist ratio, SR = LD_50_ NP/LD_50_ NP + PBO (or extract in **BOLD**). The numbers to the right of the SR in **BOLD** indicate the pepper-specific contribution to the SR = SR with PBO + Pepper/SR with PBO alone (3.8) in ternary treatments assuming multiplicative action.

Strain	Treatment	LD_50_, ng/mg (95% CL)	LD90, ng/mg (95% CL)	SR (At LD_50_)	SR (At LD_90_)
Orlando	Natural pyrethrins (NPs)	1.53 (1.0–3.3)	10.4 (4.4–74.2)	--	--
(susceptible)	NP + PBO	0.4 (0.19–0.49)	1.29 (0.8–5.3)	3.8	8.1
	NP + PBO + BPE	0.09 (0.02–0.26)	0.61 (0.35–1.5)	17 (**4.5**)	17 (**2.1**)
	NP + PBO + piperine	0.11 (0.07–0.17)	0.62 (0.39–1.28)	13.9 (**3.7**)	16.8 (**2.1**)
	NP + PBO + SPE	0.11 (0.02–0.24)	0.51 (0.23–1.02)	13.9 (**3.7**)	20.4 (**2.5**)
	NP + PBO + α-HS	0.18 (0.1–0.29)	0.82 (0.45–3.38)	8.5 (**2.2**)	12.7 (**1.6**)
Puerto Rico	Natural pyrethrins (NPs)	20 (12–24)	50.9 (33.9–107)	--	--
(resistant)	NP + PBO	3.4 (0.5–3.32)	11.5 (3.3–43.8)	5.4	4.4
	NP + PBO + BPE	2.2 (1.2–6.4)	12.7 (3.1–21.1)	9.1 (**1.7**)	4 (**0.9**)
	NP + PBO + piperine	2.1 (1–3.3)	6.3 (4.1–12.1)	9.5 (**1.8**)	8 (**1.8**)
	NP + PBO + SPE	4.8 (3.5–8.9)	43.6 (10.8–93.7)	4.16 (**0.77**)	1.2 (**0.3**)
	NP + PBO + α-HS	3.3 (0.5–6.4)	16.8 (9–60.2)	6.1 (**1.1**)	3 (**0.7**)

## Data Availability

The data presented in this study are available on request from the corresponding author. The data are not publicly available due to intellectual property concerns.

## Supplementary Materials

The following supporting information can be downloaded at https://www.mdpi.com/article/10.3390/insects17050510/s1. Figure S1: Effects of α-hydroxysanshool and piperine on (A) a pyrethroid-susceptible strain of Ae. aegypti (ORL) and (B) a pyrethroid-resistant strain (Puerto Rico). Statistical significance observed at the final three times points (27–30 min after application) is denoted with an asterisk (*p*-value < 0.05). While no differences in piperine potency was observed between strains, there appeared to be slight resistance to α-hydroxysanshool in the pyrethroid-resistant strain. Error bars were omitted for all treatments for clarity. Figure S2: Use-dependent axonal block by piperine and 10 Hz stimulation (A), and use-dependent axonal block and membrane depolarization by α-hydroxysanshool (B). Results indicate possible differential toxicodynamics with axonal ion channels.
