# Supplementary material for: Pepper Constituents Enhance the Toxicity and Neurophysiological Effects of Natural Pyrethrins in Insects
Source: Insects. 2026 May 17;17(5):510. doi: 10.3390/insects17050510 (PMC13207443; doi:10.3390/insects17050510)
Supplement: Supplementary file 1 [file insects-17-00510-s001.zip › insects-4204628-supplementary.pdf]

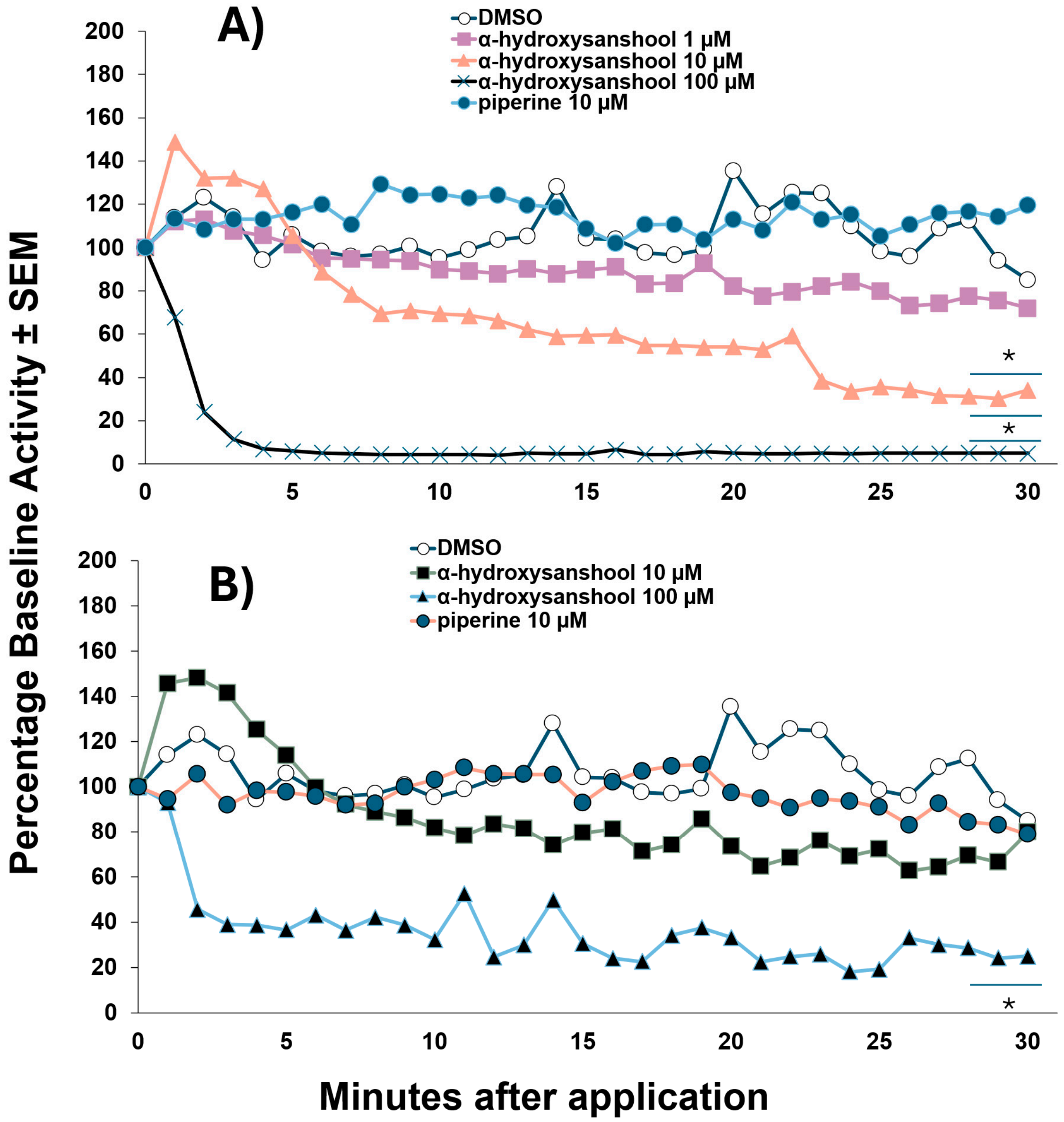

Supplemental Figure S1. Effects of α-hydroxysanshool and piperine on A) a pyrethroid-susceptible strain of Ae. aegypti (ORL) and B) a pyrethroid-resistant strain (Puerto Rico). Statistical significance observed at the final three times points (27-30 minutes after application) is denoted with an asterisk (p-value < 0.05). While no differences in piperine potency was observed between strains, there appeared to be slight resistance to α-hydroxysanshool in the pyrethroid-resistant strain. Error bars were omitted for all treatments for clarity.

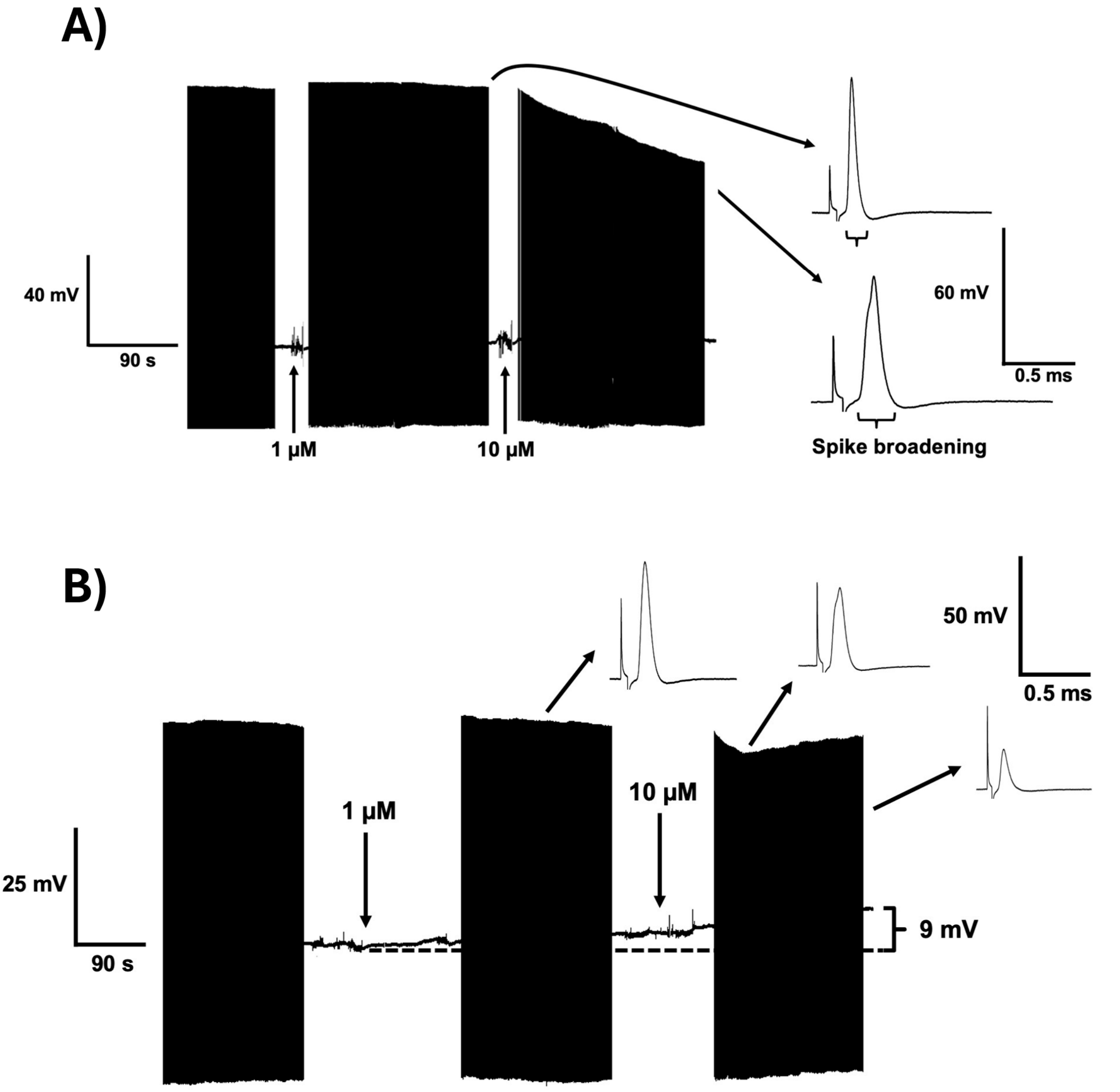

Supplemental Figure S2. Use-dependent axonal block by piperine and 10 Hz stimulation (A), and use-dependent axonal block and membrane depolarization by α-hydroxysanshool (B). Results indicate possible differential toxicodynamics with axonal ion channels.
